# Practical application and validation of the 2018 ATS/ERS/JRS/ALAT and Fleischner Society guidelines for the diagnosis of idiopathic pulmonary fibrosis

**DOI:** 10.1186/s12931-021-01670-7

**Published:** 2021-04-26

**Authors:** Angela R. Shih, Chayanin Nitiwarangkul, Brent P. Little, Benjamin W. Roop, Sreyankar Nandy, Margit V. Szabari, Nathaniel Mercaldo, Sarah Mercaldo, Sydney B. Montesi, Ashok Muniappan, Sarita R. Berigei, David A. Lynch, Amita Sharma, Lida P. Hariri

**Affiliations:** 1 Department of Pathology, Massachusetts General Hospital, 55 Fruit St, Boston, MA 02114 USA; 2 Department of Radiology, Massachusetts General Hospital, Boston, MA USA; 3 Division of Pulmonary and Critical Care Medicine, Massachusetts General Hospital, Boston, MA USA; 4 Division of Thoracic Surgery, Massachusetts General Hospital, Boston, MA USA; 5 Harvard Medical School, Boston, MA USA; 6 Department of Radiology, National Jewish Health, Denver, CO USA; 7 Department of Diagnostic and Therapeutic Radiology, Ramathibodi Hospital, Mahidol University, Bangkok, Thailand

**Keywords:** Interstitial lung disease, Lung fibrosis, Pulmonary fibrosis, Radiology and other imaging

## Abstract

**Background:**

Accurate diagnosis of idiopathic pulmonary fibrosis (IPF) is essential to inform prognosis and treatment. In 2018, the ATS/ERS/JRS/ALAT and Fleischner Society released new diagnostic guidelines for usual interstitial pneumonitis (UIP)/IPF, adding Probable UIP as a CT category based on prior studies demonstrating this category had relatively high positive predictive value (PPV) for histopathologic UIP/Probable UIP. This study applies the 2018 ATS/ERS/JRS/ALAT and Fleischner Society guidelines to determine test characteristics of CT categories in academic clinical practice.

**Methods:**

CT and histopathology were evaluated by three thoracic radiologists and two thoracic pathologists. Comparison of consensus categorization by the 2018 ATS and Fleischner Society guidelines by CT and histopathology was performed.

**Results:**

Of patients with CT UIP, 87% (PPV, 95% CI: 60–98%) had histopathologic UIP with 97% (CI: 90–100%) specificity. Of patients with CT Probable UIP, 38% (PPV, CI: 14–68%) had histopathologic UIP and 46% (PPV, CI: 19–75%) had either histopathologic UIP or Probable UIP, with 88% (CI: 77–95%) specificity. Patients with CT Indeterminate and Alternative Diagnosis had histopathologic UIP in 27% (PPV, CI: 6–61%) and 21% (PPV, CI: 11–33%) of cases with specificities of 90% (CI: 80–96%) and 25% (CI: 16–37%). Interobserver variability (kappa) between radiologists ranged 0.32–0.81.

**Conclusions:**

CT UIP and Probable UIP have high specificity for histopathologic UIP, and CT UIP has high PPV for histopathologic UIP. PPV of CT Probable UIP was 46% for combined histopathologic UIP/Probable UIP. Our results indicate that additional studies are needed to further assess and refine the guideline criteria to improve classification performance.

**Supplementary Information:**

The online version contains supplementary material available at 10.1186/s12931-021-01670-7.

## Introduction

Idiopathic pulmonary fibrosis (IPF) is a progressive, often fatal interstitial lung disease (ILD), characterized by a usual interstitial pneumonitis (UIP)-pattern on chest computed tomography (CT) scan and/or histopathologic examination of surgical biopsies [1–3]. Accurate diagnosis of UIP/IPF is paramount to inform prognosis, guide therapeutic decision-making, and determine eligibility for clinical trials. [1–3].

The ATS/ERS/JRS/ALAT and Fleischner Society separately published updated guidelines for UIP/IPF diagnosis in 2018 [2, 3]. Both guidelines effected very similar changes to CT categorization, expanding the prior system of three categories (UIP, Possible UIP, and Inconsistent with UIP patterns) to the current system of four categories (UIP, Probable UIP, Indeterminate for UIP, and Alternate Diagnosis). The main change was replacing the Possible UIP pattern with the two categories of Probable and Indeterminate for UIP, with Probable UIP defined as a subpleural-predominant reticular pattern with peripheral traction bronchiectasis and an absence of honeycombing or features to suggest an alternative diagnosis. The descriptions and criteria for the four categories were modified for CT and histopathology to better stratify cases based on the confidence level of UIP diagnosis, and therefore, better inform the confidence of IPF diagnosis.

Previous studies showed that CT Probable UIP has relatively high positive predictive value (PPV) for histopathologic UIP/Probable UIP as compared with CT categories Indeterminate for UIP and Inconsistent with UIP [4–8]. However, these studies were conducted prior to the publication of the 2018 guidelines and reported variability in test characteristics depending on institutional disease prevalence and/or potential cohort sampling bias from use of IPF trial data [4–8]. The goal of this study was to assess the practical application of the 2018 ATS/ERS/JRS/ALAT and Fleischner Society guidelines for UIP/IPF diagnosis to determine the predictive value of CT imaging for histopathologic UIP at a quaternary care hospital.

## Methods

This study was approved by the Massachusetts General Hospital Institutional Review Board (Protocol #: 2017P000176). To identify patients who had undergone surgical lung wedge biopsy for ILD evaluation, a natural language search of the pathology archives was performed for “lung interstitial fibrosis” between 2000 and 2018 at the Massachusetts General Hospital, which is a large academic institution that serves as a specialized referral center for interstitial lung disease. A total of 497 patients with surgical pathology specimens were identified from this search (Additional file 1: Figure S1). Exclusion criteria included unavailability of diagnostic high-quality CT imaging within 3 months of surgical biopsy (3 cases), unavailability of histopathology slides for review (10 cases), resections performed for neoplastic tumor excisions (226 cases), pneumonectomy specimens with an established diagnosis or end-stage fibrosis (45 cases), transbronchial biopsy specimens (94 cases) and cryobiopsy specimens (18 cases). The remaining 101 patients (85 surgical wedge biopsies and 16 pneumonectomies) were included in the study (Additional file 1: Figure S1). The cut-off year of 2018 was specifically chosen since the 2018 ATS and Fleischner Society guideline criteria may have led to clinical practice changes in surgical lung biopsy referral for patients with CT Probable UIP [2, 3].

Patient demographic data and smoking history were recorded (Table 1). CT scans were independently evaluated by three thoracic radiologists [A.S., B.L., C.N.], blinded to clinical and pathological data. Reader 1 has 20 years of experience, Reader 2 had 10 years of experience, and Reader 3 had 2 years of experience post-residency training. Discrepancies were resolved by consensus, and the consensus diagnosis was used as the comparator against histopathology. Histopathology was evaluated by consensus of two board-certified pathologists [L.P.H. and A.R.S.], blinded to clinical and radiographic data. Radiologic and pathologic interpretations were performed retrospectively and a classification of UIP, Probable UIP, Indeterminate for UIP, or Alternative Diagnosis was assigned independently for the CT and histopathology for each case using the 2018 ATS and Fleischner Society guidelines [2, 3].

**Table 1:** Patient demographics

Sex	
Female, *n* (%)	39 (39%)
Male, *n* (%)	62 (61%)
Age, years: Mean (SD)	60.7 (11.9)
Body-mass index, kg/m^2^: Mean (SD)	29.3 (4.7)
Smoking status	
Never, *n* (%)	33 (33%)
Former, *n* (%)	59 (58%)
Current, *n* (%)	9 (9%)
Pack-years of current and former smokers: Mean (SD)	33.1 (36.2)
Lung function measurements	
Forced vital capacity, % of predicted value: Mean (SD)	68.8% (18.9%)
DLCO, % of predicted value: Mean (SD)	48.9% (19.5%)

*n* number, *DLCO* diffusing capacity of the lungs for carbon monoxide

Test characteristics, including sensitivity (e.g., Pr(CT UIP|histologic UIP)), specificity (e.g., Pr(not CT UIP| not histologic UIP)), positive predictive value (PPV; e.g., Pr(histologic UIP|CT UIP)), and negative predictive value (NPV; e.g., Pr(not histologic UIP|not CT UIP)) were determined for all CT categories as compared to histopathology. Cohen’s kappa statistic was used to quantify interrater agreement between the radiologists. To quantify the uncertainty of these estimates, 95% confidence intervals or bootstrapped 95% confidence intervals (kappa) were computed. Analyses were conducted using R 3.6.2 [Vienna, Austria]. Categorical data was summarized with frequency and percentage and continuous data with mean ± standard deviation.

## Results

Of the 101 patients in our cohort (Table 1), 61% were male and 67% were former or current smokers. Average age at time of CT was 61 years (± 12 years; range: 20–84). In the cohort, 100 patients had thin section CT with slice thickness ≤ 1.5 mm, and 1 patient had high quality images with 3 mm slice thickness. CT consensus classifications were 14.9% UIP, 12.9% Probable UIP, 9.9% Indeterminate for UIP, and 62.3% Alternative Diagnosis. Histopathology classifications were 33.7% UIP, 6.9% Probable UIP, 25.7% Indeterminate for UIP, and 33.7% Alternative Diagnosis. Comparison of CT and histopathology categorization is presented in Table 2. In total, 63 cases were categorized as Alternative Diagnosis by radiology, and 34 cases were categorized as Alternative Diagnosis by histopathology, with the most common alternative diagnosis being chronic hypersensitivity pneumonitis (Additional file 2: Tables S1 and S2).

**Table 2:** Comparison of categorization between CT and histopathology

	Histopathology				
	UIP	Probable UIP	Indeterminate for UIP	Alternative Diagnosis	Total (CT** Classification)**
CT					
UIP	13	1	1	0	15
Probable UIP	5	1	4	3	13
Indeterminate for UIP	3	0	6	1	10
Alternative Diagnosis	13	5	15	30	63
Total (Histopathology Classification)	34	7	26	34	101

The test characteristics of CT categories for diagnosis of histopathologic UIP are summarized in Table 3. Of the patients with UIP by CT, 87% (95% CI: 60–98%) had UIP by histopathology with 97% (90–100%) specificity. Of patients with CT Probable UIP, 38% (14–68%) had histopathologic UIP with 88% (78–95%) specificity. Patients with CT Indeterminate for UIP and Alternative Diagnosis had histopathologic UIP in 27% (6–61%) and 21% (11–33%) of cases with specificities of 90% (80–96%) and 25% (16–37%), respectively.

**Table 3:** Test characteristics of CT categories for histopathologic UIP

	UIP	Probable UIP	Indeterminate for UIP	Alternative diagnosis
PPV	0.87 (0.60, 0.98)	0.38 (0.14, 0.68)	0.27 (0.06, 0.61)	0.21 (0.11, 0.33)
NPV	0.76 (0.65, 0.84)	0.67 (0.56, 0.77)	0.66 (0.55, 0.76)	0.45 (0.29, 0.62)
Sensitivity	0.38 (0.22, 0.56)	0.15 (0.05, 0.31)	0.09 (0.02, 0.24)	0.38 (0.22, 0.56)
Specificity	0.97 (0.90, 1.00)	0.88 (0.78, 0.95)	0.90 (0.80, 0.96)	0.25 (0.16, 0.37)

Test characteristics, including positive predictive value (PPV; e.g., Pr(histologic UIP|CT UIP)), negative predictive value (NPV; e.g., Pr(not histologic UIP|not CT UIP)), sensitivity (e.g., Pr(CT UIP|histologic UIP)), and specificity (e.g., Pr(not CT UIP| not histologic UIP)) were determined for all CT categories against histopathologic UIP. To quantify the uncertainty of these estimates, 95% confidence intervals were computed

The test characteristics of CT categories for diagnosis of histopathologic UIP or Probable UIP are summarized in Table 4. Of the patients with UIP by CT, 93% (68–100%) had histopathologic UIP or Probable UIP with 98% (91–100%) specificity. Of patients with CT Probable UIP, 46% (19–75%) had histopathologic UIP or Probable UIP with 88% (77–95%) specificity. Patients with CT Indeterminate for UIP and Alternative Diagnosis had histopathologic UIP or Probable UIP in 30% (7–65%) and 29% (18–41%) of cases with specificities of 88% (77–95%) and 25% (15–38%), respectively.

**Table 4:** Test characteristics of CT categories for diagnosis of histopathologic UIP or Probable UIP

	UIP	Probable UIP	Indeterminate for UIP	Alternative diagnosis
PPV	0.93 (0.68, 1.00)	0.46 (0.19, 0.75)	0.30 (0.07, 0.65)	0.29 (0.18, 0.41)
NPV	0.69 (0.58, 0.78)	0.60 (0.49, 0.71)	0.58 (0.47, 0.68)	0.39 (0.24, 0.57)
Sensitivity	0.34 (0.20, 0.51)	0.15 (0.06, 0.29)	0.07 (0.02, 0.20)	0.44 (0.28, 0.60)
Specificity	0.98 (0.91, 1.00)	0.88 (0.77, 0.95)	0.88 (0.77, 0.95)	0.25 (0.15, 0.38)

Test characteristics, including positive predictive value, negative predictive value, sensitivity, and specificity were determined for all CT categories as compared to histopathologic classification of UIP or Probable UIP (combined). To quantify the uncertainty of these estimates, 95% confidence intervals were computed

Test characteristics of CT categories for the corresponding histopathologic categories are summarized in Table 5. Of the patients with UIP by CT, 87% (60–98%) had UIP by histopathology with 97% (90–100%) specificity. Of patients with CT Probable UIP, 8% (0–36%) had histopathologic Probable UIP with 87% (79–93%) specificity. Patients with CT Indeterminate for UIP and Alternative Diagnosis had the corresponding histopathologic categorization in 60% (26–88%) and 48% (35–61%) of cases with specificities of 95% (87–99%) and 51% (38–63%), respectively.

**Table 5:** Test characteristics of CT categories for corresponding histopathology categories

	UIP	Probable UIP	Indeterminate for UIP	Alternative diagnosis
PPV	0.87 (0.60, 0.98)	0.08 (0.00, 0.36)	0.60 (0.26, 0.88)	0.48 (0.35, 0.61)
NPV	0.76 (0.65, 0.84)	0.93 (0.86, 0.97)	0.78 (0.68, 0.86)	0.89 (0.75, 0.97)
Sensitivity	0.38 (0.22, 0.56)	0.14 (0.00, 0.58)	0.23 (0.09, 0.44)	0.88 (0.73, 0.97)
Specificity	0.97 (0.90, 1.00)	0.87 (0.79, 0.93)	0.95 (0.87, 0.99)	0.51 (0.38, 0.63)

Test characteristics, including positive predictive value, negative predictive value, sensitivity, and specificity were determined for all CT categories against their corresponding histopathology category. To quantify the uncertainty of these estimates, 95% confidence intervals were computed

Interobserver variability (kappa) between the three radiologists is presented in Table 6. Overall kappa between Reader 1 and 2 was 0.33 (0.20–0.46, fair agreement), Reader 1 and 3 was 0.81 (0.72–0.91, very good agreement), and Reader 2 and 3 was 0.32 (0.20–0.44, fair agreement).

**Table 6:** Cohen’s kappa was used to quantify the interrater agreement between the radiologist readers

HRCT readers	Overall agreement (kappa)
R1-R2	0.33 (0.20, 0.45)
R1-R3	0.81 (0.72, 0.90)
R2-R3	0.32 (0.20, 0.44)

To quantify the uncertainty of these estimates, bootstrapped 95% confidence intervals were computed

**Fig. 1:**
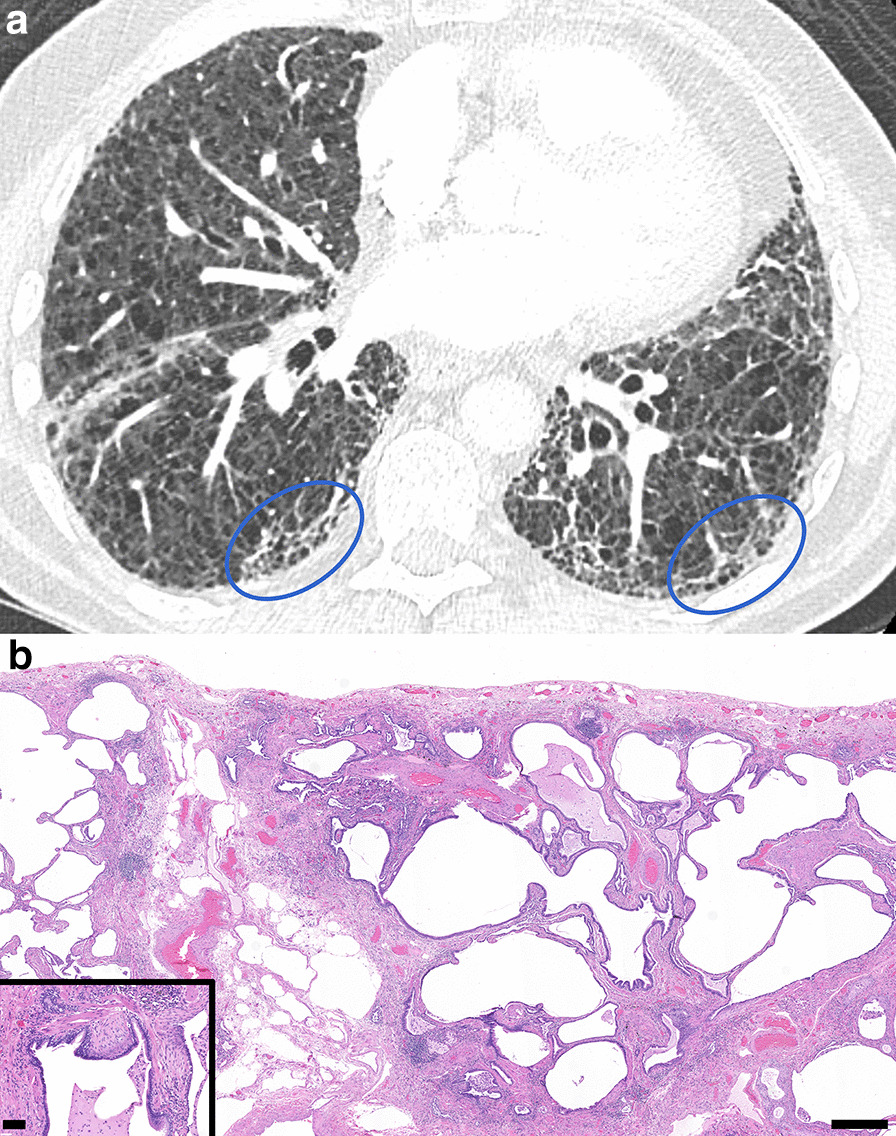
CT and Histopathology UIP. **a** Thin slice CT at the level of the right inferior pulmonary vein in a case where 2 radiologist readers independently identified honeycombing and one reader did not identify honeycombing. After discussion, the consensus interpretation amongst the radiologist readers was that honeycombing was present and the case was classified as definite UIP. The image shows all features required for categorization of UIP by the 2018 ATS and Fleischner guidelines, including basal and subpleural predominant reticulations with peripheral traction bronchiectasis and honeycombing (ovals), and an absence of features to suggest an alternative diagnosis.** b** Corresponding histopathology from a subsequent surgical lung biopsy shows all features required for categorization of UIP by both guidelines, including dense fibrosis with architectural remodeling in a patchy, subpleural distribution, with honeycombing and fibroblastic foci (inset). Hematoxylin and eosin (H&E) stain. Scalebar, panel B: 0.5 mm. Scalebar, inset: 0.1 mm

**Fig. 2:**
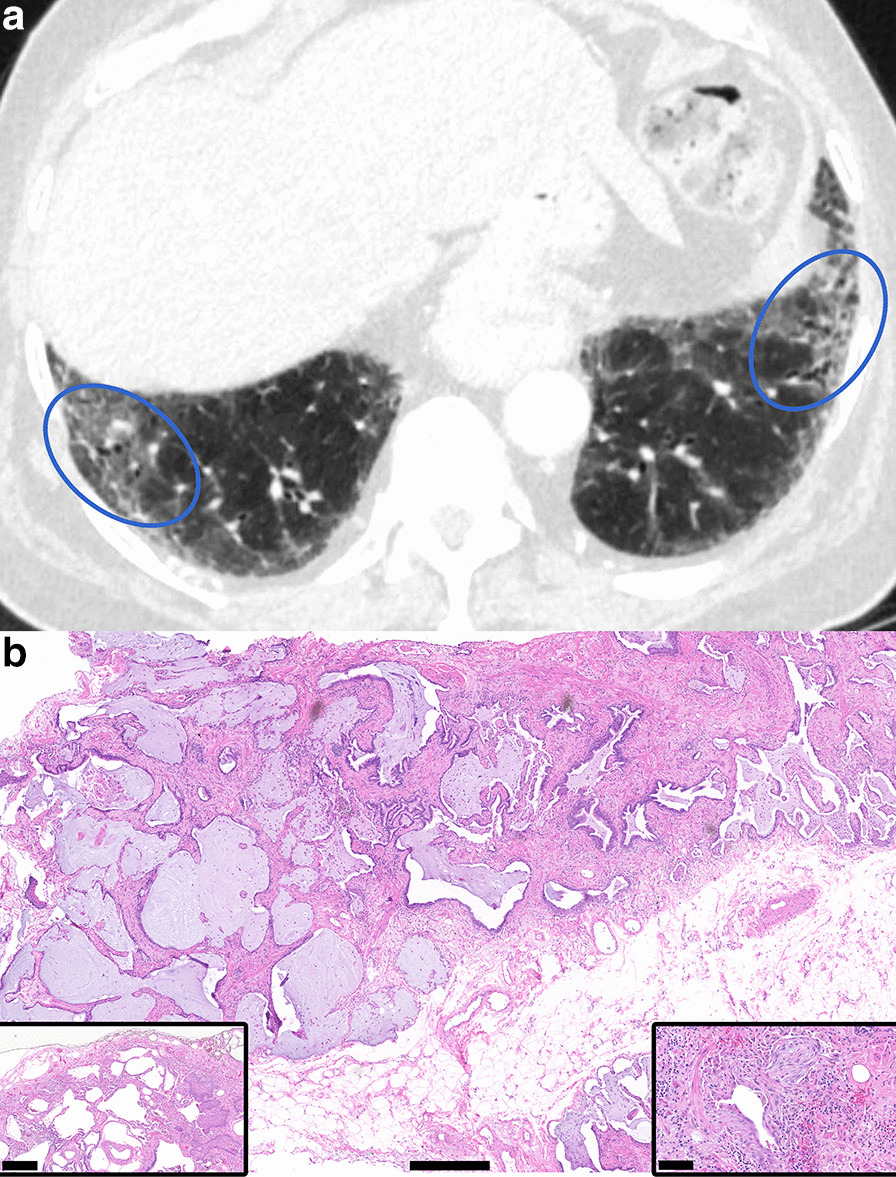
CT Probable UIP with Corresponding Histopathology UIP. **a** Thin slice CT shows basal and subpleural predominant reticulations with peripheral traction bronchiectasis (ovals) and an absence of features to suggest an alternative diagnosis, but lacks honeycombing, consistent with Probable UIP categorization by the 2018 ATS and Fleischner guidelines. The associated ground glass was interpreted as representing fine fibrosis. **b** Corresponding histopathology from a subsequent surgical lung biopsy shows all features required for categorization of UIP by both guidelines, including dense fibrosis with architectural remodeling in a patchy, subpleural distribution, with honeycombing and fibroblastic foci (right inset). Spatial heterogeneity was also present (inset, left). Hematoxylin and eosin (H&E) stain. Scalebar, panel B: 0.5 mm. Scalebar, left inset: 0.4 mm. Scalebar, right inset: 0.1 mm

**Fig. 3:**
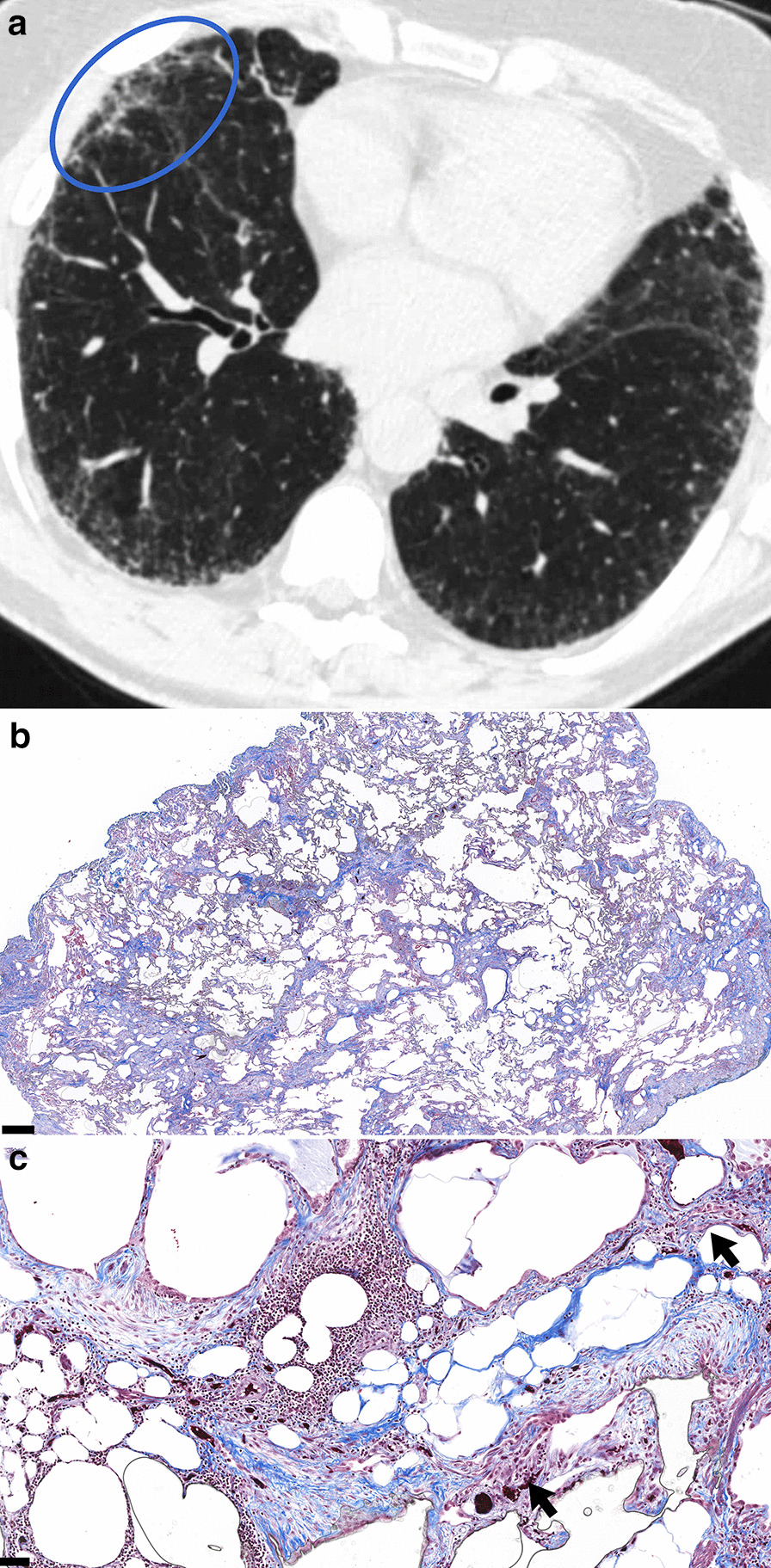
CT Probable UIP with corresponding histopathology categorized as Alternative Diagnosis. **a** Thin slice CT shows subpleural predominant reticulations without honeycombing. Two radiologist readers classified the case as Indeterminate for UIP due to absence of conspicuous traction bronchiectasis, variable distribution and an absence of features to suggest an alternative diagnosis. One radiologist reader identified traction bronchiectasis (oval). After discussion, the consensus interpretation amongst the radiologist readers was that traction bronchiectasis was present and the case was classified as Probable UIP. **b**–**c** Corresponding histopathology from the subsequent surgical lung biopsy reveals airway-centered fibrosis with patchy organizing pneumonia and peribronchiolar granulomatous inflammation (arrows), consistent with alternative diagnosis by both guidelines. The pathologic differential diagnosis included chronic hypersensitivity pneumonitis (favored) or connective-tissue related ILD. Masson’s trichrome stain. Scalebar, panel B: 0.5 mm. Scalebar, panel C: 0.1 mm

**Fig. 4:**
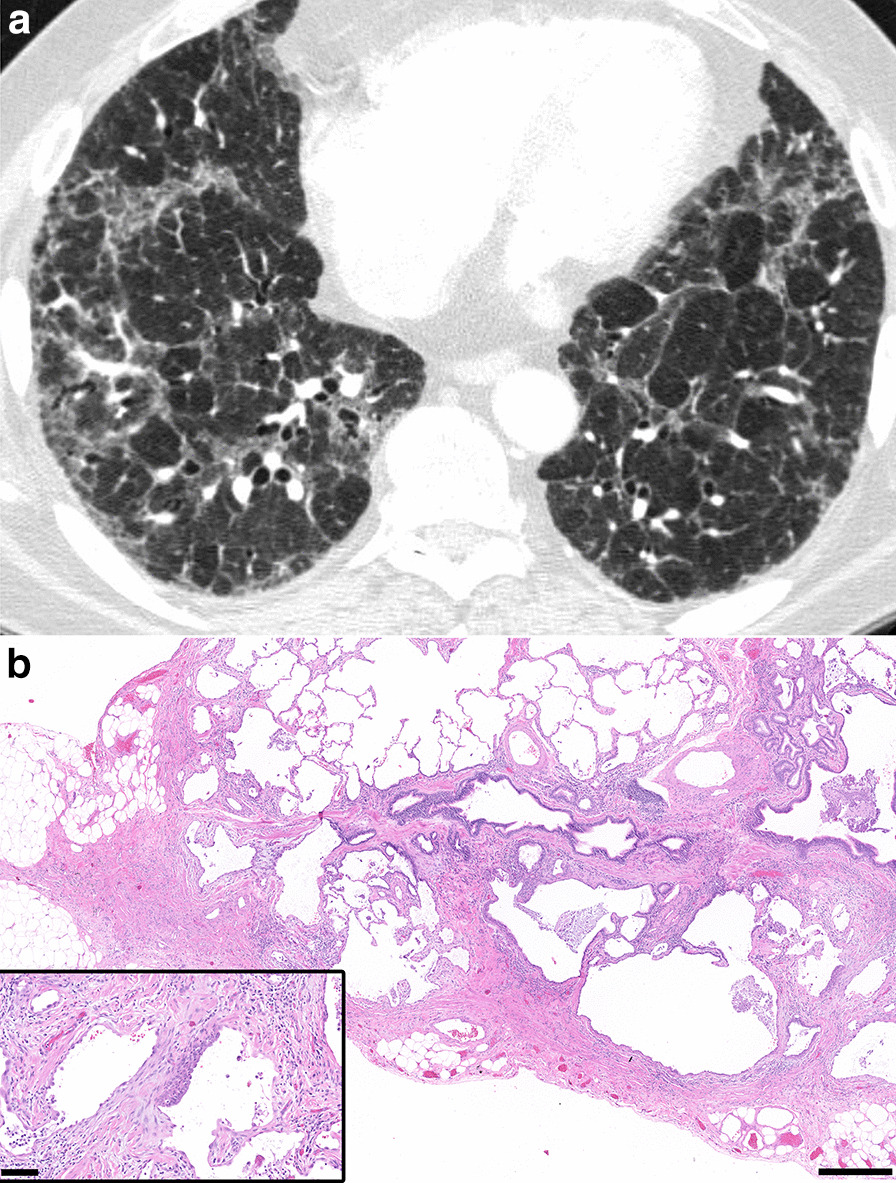
CT Alternative Diagnosis with corresponding histopathology categorized as UIP. **a** Thin slice CT demonstrates upper and mid-zone predominantly reticulation, central bronchiectasis and mosaic attenuation with lobular lucency, classified as Alternative Diagnosis categorization by the 2018 ATS and Fleischner guidelines. The radiological diagnosis was favored to be chronic hypersensitivity pneumonitis. **b** Corresponding histopathology from the subsequent surgical lung biopsy shows all features required for categorization of UIP by both guidelines, including microscopic honeycombing. There was no evidence of airway-centered fibrosis, granulomatous inflammation, or other features suggesting alternative diagnosis. Hematoxylin and eosin (H&E) stain. Scalebar, panel B: 0.5 mm. Scalebar, inset: 0.1 mm

**Fig. 5:**
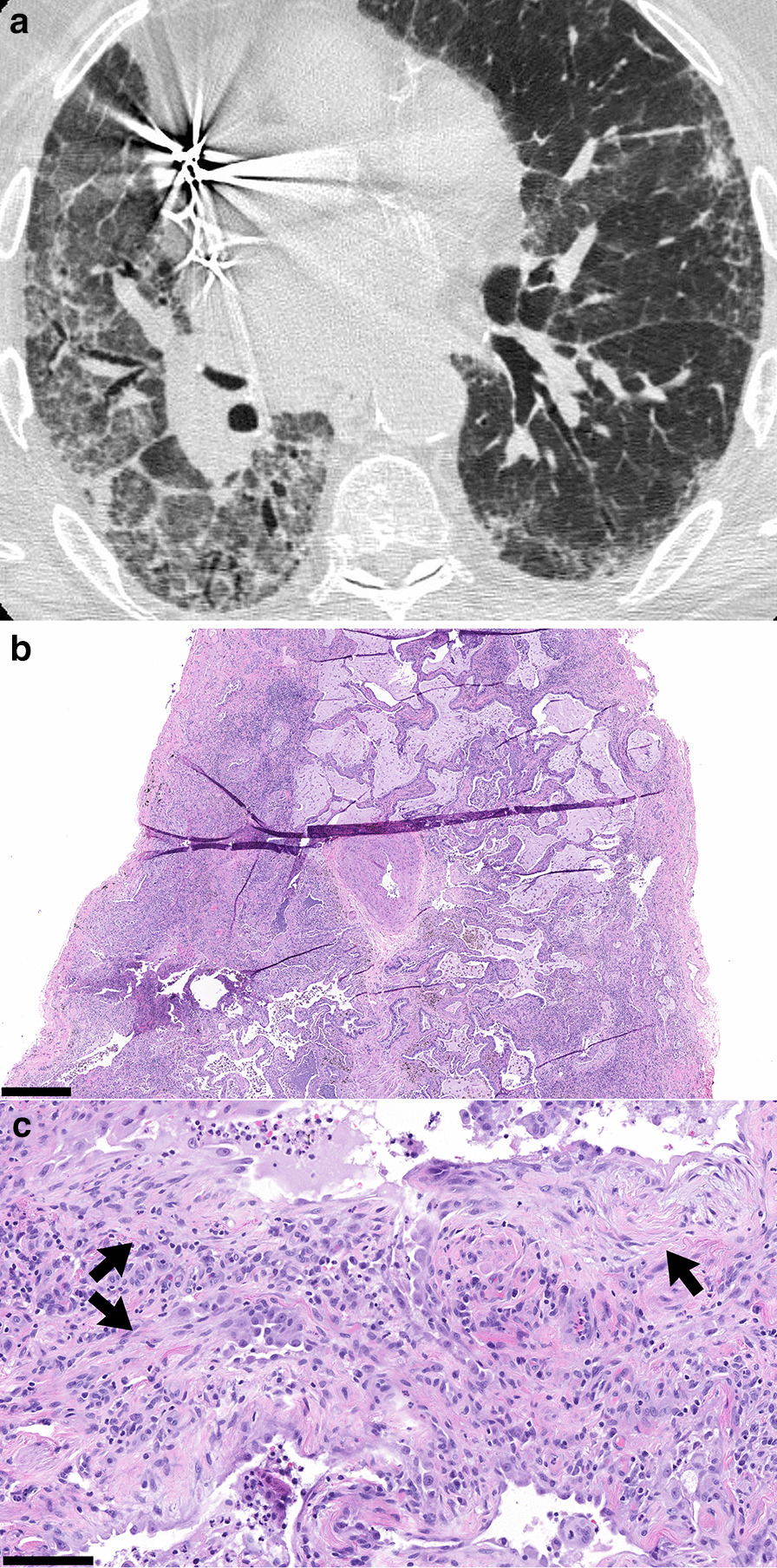
CT Alternative Diagnosis with corresponding histopathology UIP with acute exacerbation. **a** Thin slice CT shows asymmetric, non-zonal fibrosis and extensive ground glass opacity classified as Alternative Diagnosis by the 2018 ATS and Fleischner guidelines. Although the radiologic differential diagnosis included acute exacerbation of UIP, interpretation was challenging due to difficulty in classifying the underlying pattern of fibrosis as UIP **b**–**c** Corresponding histopathology from the subsequent surgical lung biopsy shows all features of UIP required by both guidelines, but with some superimposed organizing pneumonia (arrows). The amount of organizing pneumonia was not considered to be ‘prominent’, as required by the guidelines to move the classification to Alternative Diagnosis, so the case was classified as UIP but was favored to be in an accelerated stage. This case demonstrates a limitation in the application of the guidelines, where subjective features quantifiers such as ‘prominent’ result in an apparent disagreement in CT and histologic guideline categorization when the primary diagnosis is in agreement. Hematoxylin and eosin (H&E) stain. Scalebar, panel B: 0.5 mm. Scalebar, panel C: 0.1 mm

Illustrative challenging example cases of corresponding CT and surgical lung biopsy are shown in Figs. 1, 2, 3, 4, 5. Figure 1 shows a CT that was classified as definite UIP by 2 radiologist readers and Probable UIP by 1 reader due to difficulty in identifying honeycombing. After discussion, the consensus CT interpretation was definite UIP, and UIP was confirmed by histopathology. In Fig. 2, the CT shows an example of Probable UIP, which demonstrates all features required for UIP except honeycombing. There is ground glass in the region of traction bronchiectasis, which was interpreted by all 3 radiologist readers as fine fibrosis. The corresponding histopathology was categorized as UIP, documenting the presence of microscopic honeycombing. In Fig. 3, the CT was categorized as Indeterminate for UIP by 2 radiologist readers due to lack of traction bronchiectasis and variable distribution. The third radiologist reader identified traction bronchiectasis in the right middle lobe and lower lobes. After discussion, the consensus CT interpretation was Probable UIP. However, the corresponding histopathology was categorized as Alternative Diagnosis, due to the presence of conspicuous airway-centered fibrosis with peribronchiolar granulomatous inflammation. The primary diagnosis on surgical lung biopsy was most consistent with chronic hypersensitivity pneumonitis. In Fig. 4, the CT showed upper and mid-zone predominant reticulation, central bronchiectasis and mosaic attenuation with lobular lucency and was classified as Alternative Diagnosis based on the 2018 ATS and Fleischner guidelines. On the corresponding surgical lung biopsy, the histopathology was categorized as UIP. All required features of UIP were present and there were no features to suggest an alternative diagnosis, including the absence of airway-centered fibrosis and peribronchiolar granulomas. In Fig. 5, the CT demonstrated underlying fibrosis with superimposed, asymmetrical ground glass opacities. Although the radiologic differential diagnosis included UIP with acute exacerbation, the underlying pattern was not diagnostic of UIP due to an asymmetric, non-zonal fibrosis with extensive ground glass opacity; as such, a consensus classification of Alternative Diagnosis was rendered. The corresponding histopathology showed UIP features with organizing pneumonia, but the amount of organizing pneumonia was not considered to be ‘prominent’ as required by the guidelines for a classification of Alternative Diagnosis. Therefore, the case was classified as histopathologic UIP, but the primary histopathologic diagnosis was UIP in accelerated phase.

## Discussion

In this study, we applied the 2018 ATS/ERS/JRS/ALAT and Fleischner Society Guidelines for UIP/IPF diagnosis to a cohort of patients in an academic clinical practice setting and present the test characteristics for the four CT imaging categories as compared with histopathology based on the new criteria [2, 3]. Our results demonstrate high specificity and PPV for UIP by CT, which is consistent with previously published studies [4–8]. For Probable UIP by CT, specificity was high (88%), but PPV was 38% for cases with histopathologic UIP. When test characteristics for CT Probable UIP were calculated for cases categorized as either histopathologic UIP or Probable UIP, the PPV increased to 46% with a specificity of 88%. The majority of the prior studies investigating PPV of CT Probable UIP used this latter method and considered both histopathologic UIP and Probable UIP categories to be confirmed UIP [4–8]. The resulting reported PPVs ranged from 62.5 to 97.3%. However, it is unclear whether this approach is valid for the purposes of calculating test characteristics, including PPV. Cases categorized as Probable UIP by histopathology are cases whose features fall short of UIP, and therefore, are not confirmed cases of UIP.

The underlying disease prevalence affects PPV, and Brownell et al. showed that there are institutional differences in UIP/IPF prevalence [4]. The prevalence in this MGH study cohort was 33.7% UIP and 40.6% UIP/Probable UIP by histopathology. The UCSF cohort had a reported prevalence of 29% UIP/Probable UIP, while the Mayo Clinic cohort reported a prevalence of 67% UIP/Probable UIP. The PPV for Possible UIP by CT was 62.5% and 94.4% in each cohort, respectively. Similarly, many of the previously published studies had high UIP prevalence due to their use of data from patients referred for or enrolled in IPF clinical trials, or excluded subjects with other clinical diagnoses, and accordingly, the reported PPVs ranged from 81.6 to 97.3% [5–8]. Other potential sources of discordance between these studies would include inter-institutional differences by radiology and pathology groups in applying the diagnostic guideline criteria. Furthermore, it is important to note that there is sample bias in terms of which patients are referred for lung biopsy and provider/institutional referral practices for lung biopsy. Typically, patients who undergo surgical biopsy for definitive diagnosis do not have classic radiographic features, and institutions may have different thresholds at which to pursue biopsy. This inherent clinical bias in biopsy material likely has a profound impact on the variation of diagnostic prevalence of UIP between institutions.

One of the main objectives of this study was to assess the practical use of the 2018 ATS and Fleischner Society guidelines [2, 3] in an academic, non-clinical trial setting. This has highlighted some strengths and limitations in the use of the guidelines. It is important to note that the 2018 ATS and Fleischner guidelines explicitly state that the guidelines should be applied only in the clinical context of IPF [2, 3]. The addition of a fourth category to the CT categorization is a major improvement from the 2011 ATS guidelines, and facilitates better classification of cases based on confidence level. A major limitation to applying guideline criteria is the variation in feature recognition amongst readers. This limitation precedes the 2018 guidelines, but remains a continuing challenge even with the updated ATS and Fleischner guideline criteria. In particular, assessment of feature qualifiers to indicate degree (such as ‘marked’, ‘extensive’, or ‘mild’) and determination of the appropriate threshold for these qualifiers to move a case from one category to another is challenging to apply systematically and universally in practice. This is reflected in the known interobserver variability in both CT and histopathology interpretations reported in the literature, which have been moderate at best in prior studies of UIP assessment [9, 10]. In this study, two radiologist readers had unusually high agreement (kappa: 0.81, Table 6), whereas agreement with the third radiologist reader (kappas: 0.32 and 0.33, Table 6) was lower and more consistent with previously reported kappa values [9, 10]. This likely reflects closely aligned thresholds amongst the two readers with high agreement as compared to the third reader.

In the case presented in Fig. 4, the CT showed upper and mid zone predominant subpleural reticulation and central traction bronchiectasis without honeycombing. The CT also demonstrated mosaic attenuation with lobular lucency, which was determined to necessitate categorization as Alternative Diagnosis by the radiologist readers, favoring chronic hypersensitivity pneumonitis. It is known that mosaic attenuation can be seen in UIP, but there is no exact definition for the amount of mosaic attenuation that is acceptable in UIP before requiring a category change to Indeterminate for UIP or Alternative Diagnosis.

Similarly challenging situations were encountered with ground glass opacities and centrilobular nodules in evaluation of both CT and histopathology features. An illustrative example is the presence of acute exacerbation of ILD, when additional findings may make it difficult to accurately classify the underlying fibrosis, particularly if there are no prior CT scans. In the case presented in Fig. 5, the CT categorization was determined to be Alternative Diagnosis due to the presence of prominent ground glass opacities with an asymmetric and azonal distribution of fibrosis, whereas the histopathology designation was UIP because the superimposed organizing pneumonia was not considered extensive enough to classify the case as Alternative Diagnosis. Again, more specific definitions regarding assessment of features and an improved understanding of the degrees to which they may be present will be critical in improving consistency in classification. Given the subjectivity of radiologic diagnosis of UIP with current criteria, even at expert centers, it seems likely that computer based techniques will gain an increased role in establishing this diagnosis [11].

There were 15 cases in which the consensus radiology read in this study was UIP. Given that there is known interobserver variability amongst radiologists reported in the literature, it is expected that some cases categorized as UIP by the study radiologists could have been interpreted differently by the reading radiologist prior to biopsy. It is possible that there may also have been clinical details that warranted the need for biopsy. Interestingly, the test characteristics between the Probable UIP and Indeterminate for UIP CT patterns were similar in this study. However, the specific reasons for this are not readily identifiable.

The choice of comparator for calculating diagnostic test characteristics has been controversial within the ILD field. Recent publications emphasize that IPF is a multidisciplinary diagnosis (MDD), and that MDD assessment should be used as the comparator in studies calculating test characteristics [12]. However, there are some inherent issues with using MDD as the comparator. MDD depends in part on CT interpretation for its assessment, which by definition, confounds CT test characteristic analysis. Studies have also shown that there is significant interobserver variability in MDD assessments when different MDD groups assess the same patient data [10, 13]. However, the use of surgical lung biopsy as the comparator is not without its own limitations. Surgical lung biopsy interpretation also has known interobserver variability [10], and can also suffer from sampling error [14]. However, it provides an independent comparator against CT for the assessment of UIP. For these reasons, this study used histopathologic diagnosis as the comparator, which is the same method utilized by many of the prior studies that calculated CT test characteristics for UIP [4–8]. Future studies that compare CT categorization to long-term clinical course and outcome in these patient cohorts would be informative.

## Conclusions

In this study, we apply the 2018 ATS and Fleischner guidelines for UIP/IPF diagnosis and present the test characteristics for the CT imaging categories as compared with histopathology in the setting of a non-clinical trial, academic practice. To our knowledge, this is the first study to assess the test characteristics of the 2018 ATS and Fleischner guideline recommendations since their publication. We demonstrate that CT UIP and Probable UIP have high specificity for histopathologic UIP, and CT UIP has high PPV for histopathologic UIP. In our cohort, PPV of CT Probable UIP (46%) was not as high as reported in prior studies for combined histopathologic UIP/Probable UIP, which could be attributable to variability in interpretation of radiographic and histologic findings as well as the prevalence of UIP at our institution. Additional studies to assess the PPV of CT Probable UIP outside of the setting of clinical trials with long-term patient outcome will be needed in order to improve classification. Modification or refinement of guideline criteria, specifically modification of current feature qualifiers such as ‘prominent’ and ‘marked’, may also be helpful. The use of a semi-quantitative grading system, for example, may improve the practical application of the guidelines and increase reproducibility of guideline application between individual readers.

## Supplementary Information

**Additional file 1: Figure S1.** Case Inclusion and Exclusion Flowchart. A natural language search of the pathology archives between 2000 and 2018 for “lung interstitial fibrosis” yielded 497 cases, of which a total of 383 cases were excluded due to specimen type (transbronchial or cytobiopsies, pneumonectomies with an established diagnosis or end-stage fibrosis, or surgical resections for mass or tumors). Of the remaining 114 possible cases, 3 cases were excluded due to unavailability of adequate HRCT imaging within the 3 months prior to the surgical lung biopsy, and 10 cases were excluded due to unavailability of histopathology slides. In total, 101 cases were included and reviewed in the study cohort.

**Additional file 2: Table S1.** Assessment of Cases Categorized as Alternative Diagnosis. The leading diagnosis for cases categorized as Alternative Diagnosis either radiology (*n* = *63*) or histopathology (*n* = *34)* are listed. In the majority of cases, chronic hypersensitivity pneumonitis was the leading diagnosis. **Table S2.** Cases Categorized as Alternative Diagnosis by Radiology or Pathology. All cases categorized as Alternative Diagnosis either radiology (*n* = *63*) or histopathology (*n* = *34)* are listed, including the leading diagnosis (if classified as Alternative Diagnosis) and the corresponding classification by the other modality. UIP: usual interstitial pneumonitis; HP: hypersensitivity pneumonitis; NSIP: non-specific interstitial pneumonitis; DIP: desquamative interstitial pneumonitis; CTD-ILD: connective tissue disease related interstitial lung disease; Acute interstitial pneumonia (AIP); Respiratory bronchiolitis-interstitial lung disease (RB-ILD).

## Data Availability

Data sharing is not applicable to this article, as no datasets were generated or analyzed during the current study.

## Abbreviations

ALAT: Latin American Thoracic Association
ATS: American Thoracic Society
CT: Computed tomography
DLCO: Diffusing capacity of the lungs for carbon monoxide
ERS: European Respiratory Society
H&E: Hematoxylin and eosin
HRCT: High resolution computed tomography
ILD: Interstitial lung disease
IPF: Idiopathic pulmonary fibrosis
JRS: Japanese Respiratory Society
MDD: Multidisciplinary diagnosis
NPV: Negative predictive value
PPV: Positive predictive value
SD: Standard deviation
UIP: Usual interstitial pneumonitis
