# Supplementary figures and images for: Practical application and validation of the 2018 ATS/ERS/JRS/ALAT and Fleischner Society guidelines for the diagnosis of idiopathic pulmonary fibrosis
Source: Respir Res. 2021 Apr 26;22:124. doi: 10.1186/s12931-021-01670-7 (PMC8074481; doi:10.1186/s12931-021-01670-7)

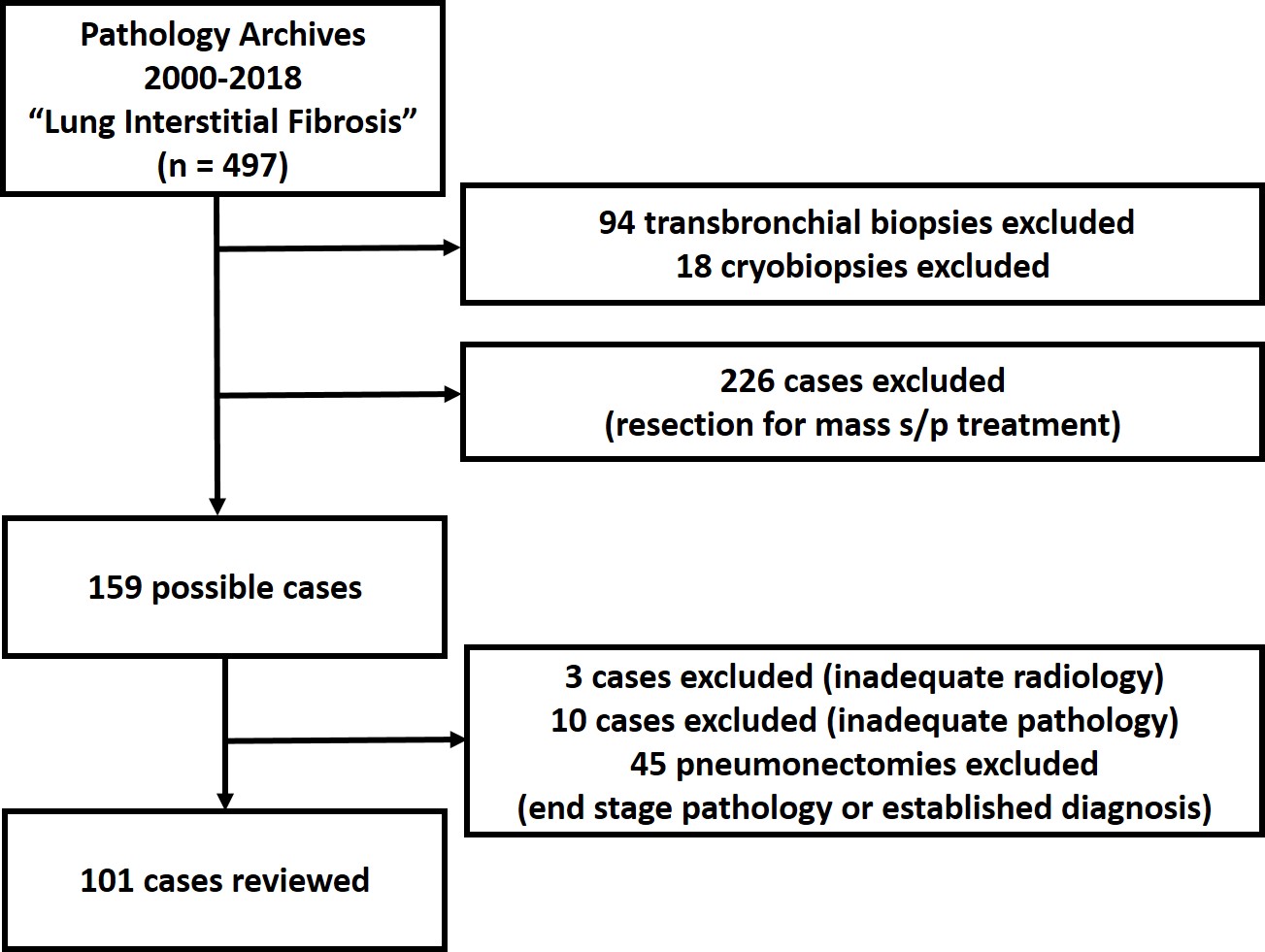

Supplement: Supplementary file 1 — Additional file 1: Figure S1. Case Inclusion and Exclusion Flowchart. A natural language search of the pathology archives between 2000 and 2018 for “lung interstitial fibrosis” yielded 497 cases, of which a total of 383 cases were excluded due to specimen type (transbronchial or cytobiopsies, pneumonectomies with an established diagnosis or end-stage fibrosis, or surgical resections for mass or tumors). Of the remaining 114 possible cases, 3 cases were excluded due to unavailability of adequate HRCT imaging within the 3 months prior to the surgical lung biopsy, and 10 cases were excluded due to unavailability of histopathology slides. In total, 101 cases were included and reviewed in the study cohort. [file 12931_2021_1670_MOESM1_ESM.jpg]
