# Supplementary material for: Practical application and validation of the 2018 ATS/ERS/JRS/ALAT and Fleischner Society guidelines for the diagnosis of idiopathic pulmonary fibrosis
Source: Respir Res. 2021 Apr 26;22:124. doi: 10.1186/s12931-021-01670-7 (PMC8074481; doi:10.1186/s12931-021-01670-7)
Supplement: Supplementary file 2 — Additional file 2: Table S1. Assessment of Cases Categorized as Alternative Diagnosis. The leading diagnosis for cases categorized as Alternative Diagnosis either radiology (n = 63) or histopathology (n = 34) are listed. In the majority of cases, chronic hypersensitivity pneumonitis was the leading diagnosis. Table S2. Cases Categorized as Alternative Diagnosis by Radiology or Pathology. All cases categorized as Alternative Diagnosis either radiology (n = 63) or histopathology (n = 34) are listed, including the leading diagnosis (if classified as Alternative Diagnosis) and the corresponding classification by the other modality. UIP: usual interstitial pneumonitis; HP: hypersensitivity pneumonitis; NSIP: non-specific interstitial pneumonitis; DIP: desquamative interstitial pneumonitis; CTD-ILD: connective tissue disease related interstitial lung disease; Acute interstitial pneumonia (AIP); Respiratory bronchiolitis-interstitial lung disease (RB-ILD). [file 12931_2021_1670_MOESM2_ESM.docx]

|  | **Alternative Diagnosis**  **on Radiology (*n=63*)** | **Alternative Diagnosis**  **on Histopathology (*n=34*)** |
| --- | --- | --- |
| Chronic hypersensitivity pneumonitis (HP) | 22 | 20 |
| Chronic organizing pneumonia | 10 | 2 |
| Desquamative interstitial pneumonitis (DIP) | 6 | 0 |
| Connective tissue disease-associated ILD (CT-ILD) | 7 | 6 |
| Non-specific interstitial pneumonia (NSIP) | 6 | 5 |
| Acute interstitial pneumonia (AIP) | 1 | 0 |
| Respiratory bronchiolitis-interstitial lung disease (RB-ILD) | 1 | 1 |
| Other Alternative Diagnosis | 6 | 0 |
| Non-specific Features | 4 | 0 |

**Additional Table S1. Assessment of Cases Categorized as Alternative Diagnosis.** The leading diagnosis for cases categorized as Alternative Diagnosis either radiology (*n = 63*) or histopathology (*n= 34)* are listed. In the majority of cases, chronic hypersensitivity pneumonitis was the leading diagnosis.

| **Radiology Classification** | **Histopathology Classification** |
| --- | --- |
| Alternative Diagnosis: Chronic HP | Indeterminate for UIP |
| Alternative Diagnosis: Chronic HP | Indeterminate for UIP |
| Alternative Diagnosis: NSIP | Indeterminate for UIP |
| Alternative Diagnosis: OP +/- HP | Indeterminate for UIP |
| Probable UIP | Alternative Diagnosis: Chronic HP |
| Alternative Diagnosis: Chronic HP | Definite UIP |
| Alternative Diagnosis: Chronic OP | Definite UIP |
| Alternative Diagnosis: Chronic HP | Probable UIP |
| Alternative Diagnosis: DIP | Alternative Diagnosis: Chronic HP |
| Alternative Diagnosis: Chronic OP | Alternative Diagnosis: Chronic HP |
| Alternative Diagnosis: RB-ILD | Alternative Diagnosis: NSIP |
| Alternative Diagnosis: DIP | Indeterminate for UIP |
| Alternative Diagnosis: Chronic OP | Alternative Diagnosis: Chronic OP |
| Alternative Diagnosis: Chronic HP | Alternative Diagnosis: Chronic HP |
| Alternative Diagnosis: CTD-ILD | Indeterminate for UIP |
| Alternative Diagnosis: Chronic HP | Alternative Diagnosis: Chronic HP |
| Alternative Diagnosis: NSIP or OP with aspiration | Indeterminate for UIP |
| Alternative Diagnosis: NSIP | Alternative Diagnosis: Chronic HP |
| Alternative Diagnosis: Bronchiolitis (aspiration/infection) | Alternative Diagnosis: CTD-ILD |
| Alternative Diagnosis: Chronic OP | Alternative Diagnosis: CTD-ILD |
| Alternative Diagnosis: Chronic HP | Alternative Diagnosis: Chronic HP |
| Alternative Diagnosis: NSIP | Alternative Diagnosis: NSIP |
| Alternative Diagnosis: Chronic HP | Alternative Diagnosis: Chronic HP |
| Alternative Diagnosis: CTD-ILD (with chronic OP) | Definite UIP |
| Alternative Diagnosis: DIP | Alternative Diagnosis: CTD-ILD |
| Alternative Diagnosis: Chronic OP or NSIP | Alternative Diagnosis: NSIP |
| Alternative Diagnosis: Chronic HP | Alternative Diagnosis: Chronic HP |
| Alternative Diagnosis: Chronic OP | Probable UIP |
| Alternative Diagnosis: Acute pneumonitis (hemorrhage versus infection) | Alternative Diagnosis: NSIP |
| Alternative Diagnosis: DIP | Definite UIP |
| Indeterminate for UIP | Alternative Diagnosis: NSIP |
| Alternative Diagnosis: Chronic HP vs fibrotic NSIP from CTD-ILD | Definite UIP |
| Alternative Diagnosis: Chronic HP | Alternative Diagnosis: Chronic HP |
| Alternative Diagnosis: Chronic OP | Indeterminate for UIP |
| Alternative Diagnosis: Chronic HP | Indeterminate for UIP |
| Alternative Diagnosis: DIP | Alternative Diagnosis: Chronic HP |
| Alternative Diagnosis: NSIP | Alternative Diagnosis: Chronic HP |
| Alternative Diagnosis: NSIP (with OP) | Alternative Diagnosis: Chronic HP |
| Alternative Diagnosis: NSIP (with acute exacerbation) | Definite UIP |
| Alternative Diagnosis: AIP | Probable UIP |
| Alternative Diagnosis: CTD-ILD | Alternative Diagnosis: Chronic HP |
| Alternative Diagnosis: Chronic OP with acute hemorrhage or infection | Indeterminate for UIP |
| Alternative Diagnosis: Chronic HP | Definite UIP |
| Alternative Diagnosis: CTD-ILD | Indeterminate for UIP |
| Alternative Diagnosis: Chronic OP | Indeterminate for UIP |
| Alternative Diagnosis: Chronic HP | Definite UIP |
| Alternative Diagnosis: Chronic HP | Indeterminate for UIP |
| Alternative Diagnosis: CTD-ILD (with chronic OP) | Definite UIP |
| Probable UIP | Alternative Diagnosis: CTD-ILD |
| Alternative Diagnosis: CTD-ILD (with chronic OP) | Definite UIP |
| Alternative Diagnosis: Chronic HP | Alternative Diagnosis: Chronic HP |
| Alternative Diagnosis: Acute OP (? Aspiration/pneumonia) | Indeterminate for UIP |
| Alternative Diagnosis: DIP | Definite UIP |
| Alternative Diagnosis: Chronic HP | Definite UIP |
| Alternative Diagnosis: Chronic OP | Probable UIP |
| Alternative Diagnosis: Chronic HP | Alternative Diagnosis: CTD-ILD |
| Alternative Diagnosis: Chronic OP | Probable UIP |
| Alternative Diagnosis: Infection with scarring | Alternative Diagnosis: Chronic HP |
| Alternative Diagnosis: Chronic HP | Alternative Diagnosis: Chronic HP |
| Probable UIP | Alternative Diagnosis: Chronic OP |
| Alternative Diagnosis: Chronic OP | Alternative Diagnosis: RB-ILD |
| Alternative Diagnosis: Chronic HP | Alternative Diagnosis: Chronic HP |
| Alternative Diagnosis: Chronic HP | Alternative Diagnosis: Chronic HP |
| Alternative Diagnosis: Chronic HP | Alternative Diagnosis: Chronic HP |
| Alternative Diagnosis: Chronic HP | Indeterminate for UIP |
| Alternative Diagnosis: CTD-ILD | Alternative Diagnosis: CTD-ILD |
| Alternative Diagnosis: Diffuse alveolar damage / Acute exacerbation | Definite UIP |

**Additional Table S2. Cases Categorized as Alternative Diagnosis by Radiology or Pathology.** All cases categorized as Alternative Diagnosis either radiology (*n = 63*) or histopathology (*n= 34)* are listed, including the leading diagnosis (if classified as Alternative Diagnosis) and the corresponding classification by the other modality. UIP: usual interstitial pneumonitis; HP: hypersensitivity pneumonitis; NSIP: non-specific interstitial pneumonitis; DIP: desquamative interstitial pneumonitis; CTD-ILD: connective tissue disease related interstitial lung disease; Acute interstitial pneumonia (AIP); Respiratory bronchiolitis-interstitial lung disease (RB-ILD).
